# Motivation, Barriers, and Suggestions for Intradialytic Exercise—A Qualitative Study among Patients and Nurses

**DOI:** 10.3390/ijerph181910494

**Published:** 2021-10-06

**Authors:** Pernille Maria Wodskou, Sasha Maria Reinhardt, Marie Borring Andersen, Stig Molsted, Lone Helle Schou

**Affiliations:** 1 Department of Nursing and Nutrition, Faculty of Health, University College Copenhagen, 2200 Copenhagen N, Denmark; sare@kp.dk (S.M.R.); marie.bandersen@hotmail.com (M.B.A.); lohs@kp.dk (L.H.S.); 2 National Institute of Public Health, University of Southern Denmark, 1455 Copenhagen K, Denmark; 3 Department of Clinical Research, Nordsjællands Hospital, 3400 Hillerød, Denmark; Stig.Moelsted@regionh.dk

**Keywords:** chronic kidney disease, hemodialysis, intradialytic exercise, patient perspective, nurses’ perspective, motivation, barriers, patient involvement, qualitative study

## Abstract

*Background*: Intradialytic exercise is an effective intervention to reduce morbidity and mortality and increase quality of life among patients with chronic kidney disease undergoing dialysis. However, implementing and sustaining it in clinical practice has proved challenging. To identify how to best design an effective and sustainable intervention in clinical practice, we aimed to explore hemodialysis patients’ and nurses’ attitudes towards intradialytic exercise, including their motivation, anticipated barriers, and suggestions for the design of a proposed exercise program. *Methods*: Data were collected through qualitative semistructured interviews with patients and focus group interviews with nurses and analyzed inductively with content analysis. *Results*: Overall, patients’ and nurses’ attitudes towards intradialytic exercise were positive. Patients were motivated by their expectations about perceived benefits, such as improved quality of life and reduced musculoskeletal pain. Their main concern was triggering dialysis machine alarms and disturbing nurses. Nurses were more skeptical of intradialytic exercise and concerned about patient safety. Patients and nurses had several ideas on how to design a safe and motivating intradialytic exercise intervention. *Conclusion*: The analysis of patients’ and nurses’ experiences and attitudes generated recommendations for an intradialytic exercise program. Recommendations include individually tailored programs that are safe and that patients can do independently, continuous collaboration between patients, nurses, physicians, and physiotherapists, and educating nurses about the benefits and safety of intradialytic exercise.

## 1. Introduction

Chronic kidney disease is a global public health problem. As the incidence continues to rise, so does the need for optimal treatment of individuals who need life-sustaining hemodialysis [1]. Patients undergoing chronic dialysis have a critical symptom burden, comorbidities, and increased mortality [2], which negatively affect physical functioning [3] and quality of life [4,5]. In addition, levels of physical exercise among individuals undergoing hemodialysis are low [6,7] because they often have a variety of uncomfortable symptoms [2,8,9]. Inactivity among hemodialysis patients is associated with increased mortality [10,11], reduced quality of life [3], and declining functional capacity for activities of daily living and occupational tasks [12]. Consequences of end-stage kidney disease and hemodialysis, such as dietary and fluid restrictions and time required for dialysis, often limit patients’ lives [13,14]. Illness and treatment become barriers to physical activity in daily life, despite the positive attitudes of most patients toward physical activity and exercise [15,16]. Intradialytic exercise (IE) (exercise training during hemodialysis treatment) can increase patients’ physical activity levels without placing additional demands on them. Growing evidence documents the positive effects of intradialytic exercise on exercise capacity [2,4,17], physical functioning [17,18,19], quality of life [2,20,21] and other patient-reported outcomes [22,23], when it combines resistance training and aerobic exercise [24,25]. The benefits of exercise may also protect the patients’ functional independence and, over time, reduce the need for homecare and help from family caregivers. In addition, studies have consistently shown that IE is safe [2,4,18,26].

Studies of patients’ opinions and experiences with IE show that they view it positively, particularly when interventions include personal instruction and follow-up [27,28,29,30]. However, patients also perceive barriers that include exercise equipment [31], safety concerns [16,29,30], disease distress [30], and nursing workloads [31,32,33]. Studies have found that nurses’ attitudes towards patients’ physical activity are correlated with patients’ activity levels, i.e., patients are less physically active when their nurses believe that physical activity is unimportant, or lack the time or feel unqualified, to discuss physical activity with patients [34,35]. Some nurses think that hemodialysis patients are unmotivated or incapable of IE, and their workloads and priorities hinder patients’ exercising [27,28,33]. Lack of related knowledge and skills have also been identified as barriers to IE [33,34]. Nurses inexperienced with IE have been found to perceive more barriers to IE than nurses with IE experience [36].

Despite the evidence that hemodialysis patients benefit from IE, it is not part of standard care [19]. Implementing exercise during hemodialysis in daily clinical practice is feasible [37], but sustaining it requires continuous commitment from dialysis and medical staff and the involvement of exercise professionals, including physiotherapists. Physiotherapists in Denmark are trained to assess physical performances and limitations to develop individualized exercise programs, and they guide and motivate patients and staff by creating an “exercise culture”. However, the latter is an added cost that may be a barrier to sustained exercise [38].

Our long-term goal is to develop and implement a sustainable IE program that takes identified barriers into account. We planned an equipment-free, simple intervention lasting 30 min or less that patients could perform independently during hemodialysis after instruction from a physiotherapist. The intervention focuses on lower body strength and standing balance exercises next to the patient’s hemodialysis station to promote physical function. However, nurses’ and patients’ attitudes to IE are needed regarding all exercise modalities during dialysis. To support the final development of the intervention, implementation, and sustainability of an IE program, we wanted to explore the perspectives of patients and nurses on our planned intervention prior to testing it.

### Aim

*The study aim was to explore the attitudes of hemodialysis patients and nurses about IE, including their motivation, anticipated barriers, and suggestions for the proposed exercise program*.

## 2. Study Design and Methods

A qualitative descriptive design was used to complete interviews with patients and nurses. Patients were interviewed using individual semistructured interviews suitable for exploring individuals’ experiences and opinions [39]. Nurses were interviewed in focus groups to support a dynamic and idea-generating dialog ideal for discussing health professionals’ experiences with and opinions of IE [40,41].

### 2.1. Study Setting and Participants

Eight semistructured qualitative interviews with patients and three semistructured focus group interviews with a total of 12 nurses were conducted at dialysis clinics in the Capital Region of Denmark in urban and periurban locations. Patient interviews were conducted in May–June 2017, and nurse focus groups occurred in March–May 2019. Both patients and nurses were intentionally selected to represent patients we envisioned participating in and benefitting from IE and nurses with representative experience and gender at the dialysis clinics. Inclusion and exclusion criteria are presented in Table 1.

Patients were recruited by nurses at the limited-care hemodialysis clinic, who identified potential participants based on the inclusion and exclusion criteria, and whom they thought would and would not be motivated to IE and obtained consent for the interviewer to contact them. Nine patients were asked to participate, and eight consented: three men and five women aged 33 to 81 (median: 65.5) years who had received hemodialysis for 1–10 (median: 6.5) years. Three participants were still employed and five were retired. At the limited-care hemodialysis clinic patients are mobile and prepare for the dialysis treatment themselves, e.g., getting all the equipment ready, before the nurse inserts the needles and starts the dialysis machine. Some limited-care patients practice the entire hemodialysis process supervised by a nurse as preparation for home hemodialysis.

Participating nurses were all experienced hemodialysis nurses with 1–20 years’ experience. They were recruited through a gatekeeper who was a nurse or a researcher at the participating clinics. The recommended number of participants in focus groups is often six to twelve. Smaller groups are advised when the topic is something that is usually talked about in small groups such as routines in a work place [40]. Due to nurses’ work schedules, four nurses participated in each focus group.

### 2.2. Data Collection

All participants chose the hemodialysis clinic as the location of their interviews. To maximize participation, nurse focus groups took place in conference rooms adjacent to clinics immediately after a shift ended.

Interviews and focus groups were conducted by the first and second authors. Semi-structured interview guides were developed based on the study aims, previous research and observations in the clinic before the study began. During the interviews, the interviewer described the proposed intervention to inspire patients to talk about their opinions and suggestions for IE in a future intervention. The interviewer included questions in the latter patient interviews based on data from previous interviews to further explore topics that emerged in the analysis. Interviews lasted 9–31 min and were audio recorded and transcribed verbatim. Nurse focus group interviews were audio recorded. A moderator took notes during the first focus group.

Theoretical saturation was achieved in both patient interviews and nurse focus groups, as no new insights about the study aim emerged in the latter interviews [39,42].

### 2.3. Data Analysis

Qualitative content analysis was used for patient interviews [43,44,45]. As the purpose was to explore attitudes, a low interpretation degree was appropriate [46]. Interview transcripts were first read as a whole, and initial categories and subcategories were recorded. Transcripts were then transferred to a qualitative data processing software program (NVivo 11 (64-bit) for Windows, QSR International Pty Ltd.: Victoria, Australia). Meaning units were identified and coded into categories and subcategories. Emerging categories and subcategories were edited to avoid overlap between categories and excessive heterogeneity in individual categories [47].

In the analysis of the nurse focus groups, audio recordings were compared with field notes and included in a content analysis [42], in which the data were processed and arranged systematically to create an overview of the data.

An example of the analysis process is presented in Table 2.

### 2.4. Ethical Considerations

The study was approved by the Danish Data Protection Agency (reference number: P-2020-74) and conducted in accordance with guidelines for storing personal data, which include anonymizing all statements. Participants were informed verbally and in writing about the study. They were assured that participation was voluntary with no influence on their hemodialysis treatment (patients) or employment (nurses) and that they could withdraw participation at any time. All participants gave informed consent.

## 3. Results

Table 3 presents an overview of the categories and subcategories emerging from the data, which are described in detail below.

### 3.1. Patient Perspective

Two categories were identified: (1) motivation for intradialytic exercise and perceived barriers and (2) the intradialytic exercise program.

#### 3.1.1. Motivation for Intradialytic Exercise and Perceived Barriers

This category contains information about factors that patients felt influences their motivation for IE. Patients were motivated by their expected benefits of physical activity and discouraged by their perceived barriers. Nurses’ opinions and approval were found to both motivate and discourage patients to IE. In general, many patients thought IE was a good idea and something they would like to do. Even patients who were more skeptical about whether they could do IE said that they would like to try it and then decide whether they wanted to continue.

##### Expected Benefits of Physical Activity

Many patients described being physically active in their daily lives in terms of housework and gardening, and some participated in physical and/or social activities, such as swimming, cycling, and fitness. Patients with musculoskeletal pain experienced physical activity as having had a positive effect on their pain and expected that IE could reduce or prevent pain. One patient talked about the body getting older and experiencing pain:

“The age… it starts to affect the legs, especially the knees when I lie still. When I have been gardening, been out digging and such, it goes better when I have been active. So therefore, I think, that some activity while lying here, either cycling or doing an exercise program, that it will be be… it will be better. Because the inactivity is not good”.

Other expected benefits included increased muscle mass, physical mobility, and higher energy levels. Several patients felt it was important to maintain or improve physical function to participate in meaningful activities and enhance quality of life.

“Well, my quality of life lies on my ability to be active, and if that starts to be difficult then my quality of life goes too, so therefore I would very much like to build something that can promote activity”.

Patients also mentioned wanting to live a long and healthy life and to be in shape for a possible kidney transplant. Short-term expected benefits included preventing cramps and resting better during hemodialysis, as well as having something to do during dialysis while reserving time outside dialysis for other things.

##### Perceived Barriers to Intradialytic Exercise

The main barrier to IE was concern about triggering the hemodialysis machine alarm. Most patients had experienced triggering the machine alarm if they moved their cannulated arm at all. The concern with the machine alarm seemed to be the alarm itself and not potential reasons for the alarm, such as changes in blood pressure or dialysis flow. No patients were concerned about hypotension or cramping resulting from IE. One patient said,

“No, I get that too when I lie down. Whether you stand or lie down, you can easily lie down again. So that’s not a problem, is it?”

Patients felt that alarms inconvenienced both them and, particularly, the nurses. Several patients also had experienced varying levels of alarm sensitivity over time. They believed that the machine would sometimes allow them to do the exercises but would be set off by the slightest movement at other times, preventing physical activity. A few patients suggested having special exercises that they could do in bed on days when the machine alarmed a lot. As one patient put it:

“But then I do it (exercise) on the bed because I just have to move this hand here, and the machine starts to roar. I just tried that, so this is the day when I can’t move that arm. I also must be careful not to bend the tube because then it (the machine) will also scold”.

Patients worried about needles and tubes as potential barriers to IE. They noted that physical activity must not accidentally pull out the needles; one cannot be physically active with sharp needles inserted, and one must be careful not to clamp or tangle the tubes. Several patients mentioned the importance of keeping the cannulated arm at rest to protect needles and tubes and avoid triggering the alarm. One patient who was being dialyzed via central venous catheter felt there was no risk of damaging her intravenous access.

In addition to alarms, needles, and tubes, the most frequently reported barrier to IE was unwillingness or, as one patient phrased it, “*laziness*”. Three patients mentioned this, reporting that they were otherwise active or were not interested in “*gymnastics*”. Two participants who were still working mentioned that they lacked the energy to be physically active during hemodialysis and needed primarily to rest.

Finally, patients mentioned fatigue during hemodialysis, feeling “*poisoned*” due to their renal failure, musculoskeletal pain, the busy workloads of nurses, and concern for fellow patients as potential barriers to intradialytic exercise.

##### Nurses’ Opinions and Approval Are Important

Nurses’ opinions were important to patients’ motivation for IE. A few patients feared that nurses would be irritated and tell them to get back into bed and lie still if the machine alarmed during exercise. On the other hand, patients expressed confidence about exercising if nurses approved, e.g.,

“Interviewer: Is there anything you worry about in relation to standing up and doing exercises? Patient: Nothing… Just that they say it’s best not to get up. That’s what the nurses say. Interviewer: So, you would feel like standing up if the nurse approved? Patient: Yes, of course. No problems. Nothing at all”.

Patients disagreed as to whether the nurses should encourage patients to exercise during dialysis. Most patients thought it could motivate them, while others did not want nurses to interfere in their decision to exercise or felt that the nurses lacked time to support IE.

#### 3.1.2. The Intradialytic Exercise Program

All patients welcomed the suggestion that a physiotherapist would instruct them the first time they did IE, after which they would be required to do the exercises themselves. Patients shared opinions as to whether tablets were a good idea to illustrate the exercises or even necessary because the proposed program was simple enough to remember.

One patient commented on the timing of exercise. She did not think that it should last more than 15–30 min at the beginning of hemodialysis to avoid exercise at the end of dialysis, when fluid removal can cause hypotension.

Several patients thought leg exercises were relevant but also asked for exercises for the upper body, particularly the neck and shoulders, where they experienced pain. A patient knowledgeable about athletics said:

“If it’s blunt needles, then I think you can do shoulder rolls and neck bends and all that”.

Some patients thought exercise equipment could boost motivation and requested dumbbells, hand grip strengtheners, massage balls to roll underfoot, elastic exercise bands, bed bikes, and ball blankets to relieve muscle tension. Several types of equipment available would also allow a variety of exercises that took the limitations of hemodialysis into account. Patients suggested equipment could be stored in a box or locker like the ones in which they kept their hemodialysis equipment, allowing them to find it themselves without help from the busy nurses.

Based on prior experience with physical activity, some patients felt that the social aspect would be motivating. They suggested that patients exercise simultaneously to, as one patient put it, “*jazz each other up a little*”. One patient thought that an element of competition could heighten his motivation, and another asked for variation, “*so you don’t do the same thing every time*”.

### 3.2. Nurses’ Perspective

Three categories were identified: (1) patient-related factors, (2) nurse routines, and (3) nurses’ motivation for intradialytic exercise.

#### 3.2.1. Patient Related Factors

The nurses pointed out that patients’ health and motivation, and the physical conditions of hemodialysis treatment, would affect IE.

The nurses described patients in hemodialysis as a heterogenous group in terms of age, level of function, and morbidity. However, most patients were elderly with symptoms of chronic illness, such as chronic fatigue and poor general condition. Nurses felt that the patients’ general condition influenced their motivation for exercise. In addition, nurses considered the many restrictions patients had in daily life, particularly related to dietary and fluid intake, important to the type of physical activity each individual patient could manage and be motivated to undertake. Nurses reported that hemodialysis is “*exhausting*” and drains patients’ energy. They interpreted patients’ habitual choices to relax and sleep during the process as saving their energy for the things in life that really mattered to them.

However, nurses mentioned several factors they thought could motivate patients to be more physically active during hemodialysis: expected benefits such as feeling more energetic and less fatigued, fewer side effects such as restless legs, fewer dietary restrictions, and improved quality of life. To maintain patients’ motivation, nurses felt that the exercise program should be individualized to the patients’ physical condition, manageable, and simple. They suggested that regular conversations between patients and the physiotherapist could help patients focus on the positive effects of exercise. They also mentioned that patients could motivate each other by exercising together or competing with or against each other. Finally, nurses felt that their professional role and long-standing relationships with patients were essential to their motivation to begin and continue IE. As one nurse put it,

”They do what we ask them to do… to a large extent”.

Most hemodialysis patients have limited mobility due to the needles and tubes connecting them to hemodialysis machines. If they move a cannulated arm, the alarm is often triggered, requiring nurses to be in constant proximity. The nurses were very aware of limitations these physical conditions placed on IE. They reported that IE required “*good access*”, meaning a well-functioning fistula with blunt needles or a central or peripheral venous catheter, because sharp needles would increase the risk of vascular perforation. The space between beds is limited due to the presence of the hemodialysis machines, tubes, and power cords, requiring that care be taken when moving around. The nurses were concerned that the lack of available floor space, combined with physical exercise, could hinder their access to patients in case of an emergency. Nurses preferred IE to take place in bed with equipment such as elastic exercise bands, small ankle and hand weights, or bed bikes, with which several of the nurses had experience. Nurses also mentioned that restricted space and lack of privacy could decrease the motivation of more modest patients.

#### 3.2.2. Nurse Routines

Nurses described their work in the clinic as characterized by routines, such as starting and ending hemodialysis, checking blood test results, and administrating medications. IE would have to fit into their accustomed routines, but the degree of required fit would depend on how involved they needed to be. If the nurses were to be involved, they wanted IE to begin after they had started dialysis on all their patients, checked blood tests, and administered medications. To save time, all exercise equipment needed to be user friendly and easy to obtain and clean. The nurses felt that less involvement would be better, for example, if patients could begin their exercise independently, and its timing in relation to hemodialysis routines would matter less. Similarly, if patients had personal exercise equipment that they could keep in their lockers, nurses would not have to make time to obtain and clean equipment.

#### 3.2.3. Nurses’ Motivation for Intradialytic Exercise

In general, all nurses viewed IE positively. They agreed that exercise would contribute to patients’ physical and mental well-being and, particularly, their quality of life. These benefits were their primary motivation for supporting the intervention. However, nurses felt it was unrealistic for all patients to do IE, and some thought that patients doing it while standing posed too great a risk. As one nurse argued,

“I wouldn’t dare.” Another nurse agreed: “The standing and jumping and getting out of bed… I just can’t see it”.

Some nurses had experience with hemodialysis patients exercising by using bed bikes or participating in between-treatment “*walking teams*”, but their opinions of IE were primarily based on their knowledge of pathophysiology and the physical conditions of hemodialysis and their views on how an extra task would affect their already busy workdays. Nurses agreed that they lacked knowledge about the effects and possible negative consequences of IE and asked for information on the subject. They needed to feel certain that it would not endanger patients by, for example, overlooking contraindicating comorbidities. Therefore, they wanted the physician to approve individual patients’ participation in an exercise program.

Nurses viewed involving a physiotherapist in the development of individual IE programs as crucial to successful implementation. Specifically, they suggested that the physiotherapist, in collaboration with the patient and nurse, develop the exercise program, help the patient get well under way, and regularly follow up to adjust the program as needed. Nurses would have no role in the initial introduction, which they viewed as a barrier because of the time required. They perceived their role as primarily motivating, following up on patients’ progress, and helping to address any difficulties. One nurse noted that management support, enthusiasm among nursing colleagues, and a pilot test on a small group of patients, were needed to maintain their motivated support for IE in a busy clinic. Additionally, nurses reported that they could only give low priority to IE if unexpected events occurred in the clinic.

## 4. Discussion

Overall, patients and nurses positively viewed intradialytic exercise. They agreed that long-term expected benefits and nurses’ support would motivate patients for IE. Their mutual overall objective was improved quality of life. However, aside from an expected increase in energy and reduction of fatigue, patients and nurses differed as to the effects of exercise they expected would generate this improvement. Patients highlighted increased muscle mass, maintaining physical mobility and meaningful activities, and preventing musculoskeletal pain, while nurses focused on fewer symptoms, such as restless legs, and fewer diet restrictions in daily life. Patients, but not nurses, also mentioned expected short-term benefits, such as having something to do during hemodialysis, being able to relax better, saving time, and preventing cramps. Reviews of the perceptions of patients with chronic kidney disease on physical activity noted similar long and short-term benefits [8,16] for both exercise in general and IE. Interestingly, nearly half of participants (*n* = 9) in a UK study could not mention any possible benefit of physical activity for patients on dialysis [48]. This suggests that patients’ experiences and expectations of physical activity and exercise vary greatly, and it may be the case that patients who are willing to participate in an exercise study are those who view the intervention most favorably. Jhamb et al. [27] also found that patients and nurses agreed on the benefits of exercise. However, in a study by Young et al. [33], patients’ expectations were similar to those in our study and others [16], but nurses held very different expectations. Both qualitative and quantitative studies have demonstrated the importance of encouragement from nurses and other dialysis staff to patients’ motivation to exercise [27,34,35]. In our study, patients and nurses alike also highlighted the importance of their relationship to patients’ motivation.

Nurses viewed their role as central, and primarily related to motivation and follow up, and felt that participating actively, such as by supplying patients with exercise equipment, was untenable. Patients unanimously expressed their belief that they could do the exercises themselves without help from nurses. Jhamb et al. [27] reported that nurses viewed their role in much the same way as those in our study, while Thompson et al. [28] reported that nurses saw themselves in a more practical role, assisting patients with equipment, and leaving motivation to physicians.

Patients in our study anticipated few barriers to IE, and they could largely see ways to overcome them. The most important problem was dialysis machine alarms, which were annoying and created extra work for nurses. They addressed concerns about damaging their fistula or intravenous access by suggesting exercises that protected needles and tubes. Their unwillingness or laziness, as some patients put it, could be coped with by the motivation of fellow patients and nurses. In contrast, nurses, on the other hand, had more reservations about IE. They thought that many patients would be uninterested or unable to do IE due to chronic illness, general condition, diet restrictions and fatigue, which none of the patients mentioned. This could be because interviewed patients were in a limited care clinic, where patients are generally higher functioning than those in general dialysis clinics, while the nurses were from different dialysis clinics treating patients with a wider range of care needs. However, despite their skepticism, nurses did not reject the proposal for IE, but they emphasized the importance of tailoring the intervention to fit the desires and condition of individual patients, and of collaboration with a physiotherapist. The same concerns were found in a preintervention study by Young et al. [33]. However, the intervention in their study was limited to cycling, whereas the nurses in our study were invited to give their opinion on the design of the intervention, which can explain their agreement to give the intervention a try.

The nurses’ main concern was patient safety. They voiced concern about patients exercising next to beds and felt more comfortable with in-bed exercises. Nurses in our study emphasized the need for having and protecting good hemodialysis access, and they worried about injuries due to the lack of space in the hemodialysis clinic. Nurses also lacked sufficient knowledge about the effects, risks, and contraindications of intradialytic exercise. In a study by Regolisti et al. [34], nurses also reported a lack of knowledge of physical activity in relation to hemodialysis. The lack of knowledge of the benefits of physical activity could also be related to the low priority nurses placed on physical activity. As in studies by Thompson et al. [28] and Young et al. [33], the nurses in our study already felt busy with nursing tasks related to hemodialysis treatment, to which IE would only add. Hence, it was a low priority.

## 5. Strengths and Limitations

Only eight patients and 12 nurses participated in the study. However, data saturation occurred in both the patient interviews and nurse focus groups. Nurse focus groups were smaller than usually recommended [40], but discussions of IE were highly informative. As a nature of the protocol, interviewed patients had a relatively high level of function and received treatment at a limited care hemodialysis clinic, and their experiences and opinions are probably not representative of all hemodialysis patients. The inclusion of nurses from other hemodialysis clinics treating patients with lower levels of self-care mitigates this limitation. However, there is risk of selection bias. Our results can be used to guide the design of an intervention that can be implemented in clinical practice to maintain or enhance patients’ physical functioning, quality of life and self-reliance in everyday life.

## 6. Conclusions

This study investigated patients’ and nurses’ opinions of IE and their anticipated motivators, barriers, and suggestions for an exercise intervention. Patients and nurses were motivated by the expected benefits of IE, such as lower symptom burden and improved activity levels, resulting in increased quality of life. Patient barriers were predominantly concerns about the dialysis machine alarm disturbing the nurses and protecting needles and fistulas while nurses were concerned about patient safety in general. Both patients and nurses had several suggestions to improve the proposed IE program. The resulting recommendations support implementation of an intradialytic exercise intervention.

- An exercise program should be collaboratively individualized by the patient, nurse, physician, and physiotherapist to ensure patient safety and fit the patient’s ability and motivation.
- Intradialytic exercise should fit into existing nursing routines and require minimal nursing involvement.
- Due to patient safety and the lack of floor space, exercise should take place in or immediately next to the bed.
- Equipment should be hygienic, preferably personal, available directly to patients, and kept in their lockers.
- A physiotherapist should instruct patients at the beginning of an intradialytic exercise program and follow up regularly.
- Nursing staff should be educated about the benefits and safety of intradialytic exercise.
- Highly motivated nurses should be engaged to act as champions and motivate colleagues.

Further studies should address the feasibility and efficacy of intradialytic exercise intervention based on these recommendations and investigate its implementation and maintenance in hemodialysis clinics over time.

## Figures and Tables

**Table 1:** Inclusion and exclusion criteria for patients and nurses.

	Inclusion Criteria	Exclusion Criteria
**Patients**	Received hemodialysis in the clinic for a minimum of 3 months Age > 18 years Able to walk without aids Able to have a conversation in Danish without difficulty Able to give informed consent Able to reflect and share their thoughts on the topic in a coherent and relevant way	Severe mental illness Cognitive disorders, e.g., dementia Crisis Lower extremity amputation Severe polyneuropathy (unable to feel own legs) Blindness or severe visual impairment impeding safety during physical activity Comorbidity preventing physical exercise at moderate intensity, e.g., low ejection fraction. Cannulation with sharp needles
**Nurses**	Working as a nurse in the dialysis clinic for at least 6 months	None

**Table 2:** Example of the analysis process.

Meaning Units	Code	Sub-Category	Category
Interviewer: *Do you have any concerns about IE? Do you think that anything bad could happen*? Patient: *No, no, only that the machine would alarm all the time, because you move the arm, right*.	Dialysis machine alarming	Barriers to IE	Motivation for IE and perceived barriers
*Then I think: I wonder if my laziness starts? But I think it’s a good idea, I do. I probably need a little push to get started. But then we have the nurses.*	Nurses as motivators	Nurses’ opinions	Motivation for IE and perceived barriers
*And then it should be varied, so that you don’t do the same (exercises) every time.*	Variation		The intradialytic exercise program

**Table 3:** Overview of findings.

	Categories and Sub-Categories
**Patients**	Motivation for intradialytic exercise and perceived barriers ▪ Expected benefits of physical activity ▪ Barriers to intradialytic exercise ▪ Nurses’ opinions and approval are important The intradialytic exercise program
**Nurses**	Patient-related factors Nurse routines Nurses’ motivation for intradialytic exercise

## Data Availability

The data used during the current study are available from the corresponding author on reasonable request.

## References

[1] Grassmann A., Gioberge S., Moeller S., Brown G. (2005). ESRD Patients in 2004: Global Overview of Patient Numbers, Treatment Modalities and Associated Trends. Nephrol. Dial. Transplant. Off. Publ. Eur. Dial. Transpl. Assoc.—Eur. Ren. Assoc..

[2] Pu J., Jiang Z., Wu W., Li L., Zhang L., Li Y., Liu Q., Ou S. (2019). Efficacy and Safety of Intradialytic Exercise in Haemodialysis Patients: A Systematic Review and Meta-Analysis. BMJ Open.

[3] Kaysen G.A., Larive B., Painter P., Craig A., Lindsay R.M., Rocco M.V., Daugirdas J.T., Schulman G., Chertow G.M., Group F.H.N.T. (2011). Baseline Physical Performance, Health, and Functioning of Participants in the Frequent Hemodialysis Network (FHN) Trial. Am. J. Kidney Dis. Off. J. Natl. Kidney Found..

[4] Chung Y.-C., Yeh M.-L., Liu Y.-M. (2017). Effects of Intradialytic Exercise on the Physical Function, Depression and Quality of Life for Haemodialysis Patients: A Systematic Review and Meta-Analysis of Randomised Controlled Trials. J. Clin. Nurs..

[5] De Knudsen S.P., Eidemak I., Molsted S. (2016). Health Related Quality of Life in 2002 and 2015 in Patients Undergoing Hemodialysis: A Single Center Study. Ren. Fail..

[6] Anderton N., Giri A., Wei G., Marcus R.L., Chen X., Bjordahl T., Habib A., Herrera J., Beddhu S. (2015). Sedentary Behavior in Individuals with Diabetic Chronic Kidney Disease and Maintenance Hemodialysis. J. Ren. Nutr. Off. J. Counc. Ren. Nutr. Natl. Kidney Found..

[7] Johansen K.L., Chertow G.M., Kutner N.G., Dalrymple L.S., Grimes B.A., Kaysen G.A. (2010). Low Level of Self-Reported Physical Activity in Ambulatory Patients New to Dialysis. Kidney Int..

[8] Clarke A.L., Jhamb M., Bennett P.N. (2019). Barriers and Facilitators for Engagement and Implementation of Exercise in End-stage Kidney Disease: Future Theory-based Interventions Using the Behavior Change Wheel. Semin. Dial..

[9] Delgado C., Johansen K.L. (2012). Barriers to Exercise Participation among Dialysis Patients. Nephrol. Dial. Transplant. Off. Publ. Eur. Dial. Transpl. Assoc.—Eur. Ren. Assoc..

[10] Johansen K.L., Kaysen G.A., Dalrymple L.S., Grimes B.A., Glidden D.V., Anand S., Chertow G.M. (2013). Association of Physical Activity with Survival among Ambulatory Patients on Dialysis: The Comprehensive Dialysis Study. Clin. J. Am. Soc. Nephrol. CJASN.

[11] Matsuzawa R., Matsunaga A., Wang G., Kutsuna T., Ishii A., Abe Y., Takagi Y., Yoshida A., Takahira N. (2012). Habitual Physical Activity Measured by Accelerometer and Survival in Maintenance Hemodialysis Patients. Clin. J. Am. Soc. Nephrol. CJASN.

[12] Heiwe S., Jacobson S.H. (2011). Exercise Training for Adults with Chronic Kidney Disease. Cochrane Database Syst. Rev..

[13] Makaroff K.L.S. (2012). Experiences of Kidney Failure: A Qualitative Meta-Synthesis. Nephrol. Nurs. J. J. Am. Nephrol. Nurses’ Assoc..

[14] Reid C., Seymour J., Jones C. (2016). A Thematic Synthesis of the Experiences of Adults Living with Hemodialysis. Clin. J. Am. Soc. Nephrol. CJASN.

[15] Hannan M., Bronas U.G. (2017). Barriers to Exercise for Patients with Renal Disease: An Integrative Review. J. Nephrol..

[16] Zhang J., Bennett P.N. (2019). The Perception of People with Chronic Kidney Disease towards Exercise and Physical Activity: A Literature Review. Ren. Soc. Australas. J..

[17] Sheng K., Zhang P., Chen L., Cheng J., Wu C., Chen J. (2014). Intradialytic Exercise in Hemodialysis Patients: A Systematic Review and Meta-Analysis. Am. J. Nephrol..

[18] Salhab N., Karavetian M., Kooman J., Fiaccadori E., El Khoury C.F. (2019). Effects of Intradialytic Aerobic Exercise on Hemodialysis Patients: A Systematic Review and Meta-Analysis. J. Nephrol..

[19] Milam R.H. (2016). Exercise Guidelines for Chronic Kidney Disease Patients. J. Ren. Nutr. Off. J. Counc. Ren. Nutr. Natl. Kidney Found..

[20] Young H.M.L., March D.S., Graham-Brown M.P.M., Jones A.W., Curtis F., Grantham C.S., Churchward D.R., Highton P., Smith A.C., Singh S.J. (2018). Effects of Intradialytic Cycling Exercise on Exercise Capacity, Quality of Life, Physical Function and Cardiovascular Measures in Adult Haemodialysis Patients: A Systematic Review and Meta-Analysis. Nephrol. Dial. Transplant. Off. Publ. Eur. Dial. Transpl. Assoc.—Eur. Ren. Assoc..

[21] Huang M., Lv A., Wang J., Xu N., Ma G., Zhai Z., Zhang B., Gao J., Ni C. (2019). Exercise Training and Outcomes in Hemodialysis Patients: Systematic Review and Meta-Analysis. Am. J. Nephrol..

[22] Song Y.-Y., Hu R.-J., Diao Y.-S., Chen L., Jiang X.-L. (2018). Effects of Exercise Training on Restless Legs Syndrome, Depression, Sleep Quality, and Fatigue Among Hemodialysis Patients: A Systematic Review and Meta-Analysis. J. Pain Symptom Manage..

[23] Bohm C., Schick-Makaroff K., MacRae J.M., Tan M., Thompson S. (2019). The Role of Exercise in Improving Patient-Reported Outcomes in Individuals on Dialysis: A Scoping Review. Semin. Dial..

[24] Gomes Neto M., de Lacerda F.F.R., Lopes A.A., Martinez B.P., Saquetto M.B. (2018). Intradialytic Exercise Training Modalities on Physical Functioning and Health-Related Quality of Life in Patients Undergoing Maintenance Hemodialysis: Systematic Review and Meta-Analysis. Clin. Rehabil..

[25] Ferrari F., Helal L., Dipp T., Soares D., Soldatelli Â., Mills A.L., Paz C., Tenório M.C.C., Motta M.T., Barcellos F.C. (2020). Intradialytic Training in Patients with End-Stage Renal Disease: A Systematic Review and Meta-Analysis of Randomized Clinical Trials Assessing the Effects of Five Different Training Interventions. J. Nephrol..

[26] Greenwood S.A., Koufaki P., Macdonald J.H., Bhandari S., Burton J.O., Dasgupta I., Farrington K., Ford I., Kalra P.A., Kean S. (2021). Randomized Trial—PrEscription of IntraDialytic Exercise to Improve QuAlity of Life in Patients Receiving Hemodialysis. Kidney Int. Reports.

[27] Jhamb M., McNulty M.L., Ingalsbe G., Childers J.W., Schell J., Conroy M.B., Forman D.E., Hergenroeder A., Dew M.A. (2016). Knowledge, Barriers and Facilitators of Exercise in Dialysis Patients: A Qualitative Study of Patients, Staff and Nephrologists. BMC Nephrol..

[28] Thompson S., Tonelli M., Klarenbach S., Molzahn A. (2016). A Qualitative Study to Explore Patient and Staff Perceptions of Intradialytic Exercise. Clin. J. Am. Soc. Nephrol. CJASN.

[29] Ghafourifard M., Mehrizade B., Hassankhani H., Heidari M. (2021). Hemodialysis Patients Perceived Exercise Benefits and Barriers: The Association with Health-Related Quality of Life. BMC Nephrol..

[30] Li T., Lv A., Xu N., Huang M., Su Y., Zhang B., Li X. (2021). Barriers and Facilitators to Exercise in Haemodialysis Patients: A Systematic Review of Qualitative Studies. J. Adv. Nurs..

[31] Heiwe S., Tollin H. (2012). Patients’ Perspectives on the Implementation of Intra-Dialytic Cycling—A Phenomenographic Study. Implement. Sci. IS.

[32] Thompson S., Klarenbach S., Molzahn A., Lloyd A., Gabrys I., Haykowsky M., Tonelli M. (2016). Randomised Factorial Mixed Method Pilot Study of Aerobic and Resistance Exercise in Haemodialysis Patients: DIALY-SIZE!. BMJ Open.

[33] Young H.M.L., Hudson N., Clarke A.L., Dungey M., Feehally J., Burton J.O., Smith A.C. (2015). Patient and Staff Perceptions of Intradialytic Exercise before and after Implementation: A Qualitative Study. PLoS ONE.

[34] Regolisti G., Maggiore U., Sabatino A., Gandolfini I., Pioli S., Torino C., Aucella F., Cupisti A., Pistolesi V., Capitanini A. (2018). “Esercizio fisico nel paziente con insufficienza renale cronica” of the S.I. di. Interaction of Healthcare Staff’s Attitude with Barriers to Physical Activity in Hemodialysis Patients: A Quantitative Assessment. PLoS ONE.

[35] Michou V., Kouidi E., Liakopoulos V., Dounousi E., Deligiannis A. (2019). Attitudes of Hemodialysis Patients, Medical and Nursing Staff towards Patients’ Physical Activity. Int. Urol. Nephrol..

[36] Bennett P.N., Peter J., Wang W., Street M. (2016). Attitudes of Nephrology Nurses Toward Patient Exercise During Hemodialysis. Nephrol. Nurs. J. J. Am. Nephrol. Nurses’ Assoc..

[37] Young H.M.L., Jeurkar S., Churchward D.R., Dungey M., Stensel D.J., Bishop N.C., Greenwood S.A., Singh S.J., Smith A.C., Burton J.O. (2018). Implementing a Theory-Based Intradialytic Exercise Programme in Practice: A Quality Improvement Project. Clin. Kidney J..

[38] Bennett P.N., Breugelmans L., Barnard R., Agius M., Chan D., Fraser D., McNeill L., Potter L. (2010). Sustaining a Hemodialysis Exercise Program: A Review. Semin. Dial..

[39] Kvale S., Brinkmann S. (2015). Interview.

[40] Halkier B. (2008). Fokusgrupper (Focus Groups).

[41] Krueger R.A., Casey M.A. (2000). Focus Groups: A Pracitical Guide for Applied Research.

[42] Glaser B.G., Strauss A.L. (1967). The Discovery of Grounded Theory: Strategies for Qualitative Research.

[43] Elo S., Kyngäs H. (2008). The Qualitative Content Analysis Process. J. Adv. Nurs..

[44] Graneheim U.H., Lundman B. (2004). Qualitative Content Analysis in Nursing Research: Concepts, Procedures and Measures to Achieve Trustworthiness. Nurse Educ. Today.

[45] Krippendorff K. (2013). Content Analysis: An Introduction to Its Methodology.

[46] Graneheim U.H., Lindgren B.-M., Lundman B. (2017). Methodological Challenges in Qualitative Content Analysis: A Discussion Paper. Nurse Educ. Today.

[47] Elo S., Kääriäinen M., Kanste O., Pölkki T., Utriainen K., Kyngäs H. (2014). Qualitative Content Analysis. SAGE Open.

[48] Sutherland S., Penfold R., Doherty A., Milne Z., Dawes H., Pugh C., Boulton M., Newton J.L. (2019). A Cross-Sectional Study Exploring Levels of Physical Activity and Motivators and Barriers towards Physical Activity in Haemodialysis Patients to Inform Intervention Development. Disabil. Rehabil..

